# The Rain for Nine Years

**Published:** 1840-11

**Authors:** 


*The Rain for nine years*—The results of Meteorological observation are highly interesting and instructive. They belong to the scientific world, and should be thrown into the general treasury of knowledge to enrich those who love to draw from an exhaustless source. The superficial may see little to attract his attention in long columns of figures, but there are minds that find food for thought among these "husks" of philosophy.

The table below has been accurately calculated from the valuable Register long kept by the Rev. Dr. Allan.—It exhibits the result of nine years observation in reference to the amount of rain which has fallen monthly at Huntsville. The calculations appended may serve to assist the mind in grasping truth well nigh beyond its scope. If they chance to shake the credulity of any, with the data given, let them test their correctness. Table to be read as inches and thousandths.

| | 1831 | 1832 | 1833 | 1834 | 1835 |
|---|---|---|---|---|---|
| January, - - - | 6,708 | 2,771 | 6,873 | 10,410 | 4,855 |
| February, - - - | 2,344 | 3,456 | 11,451 | 8,242 | 3,186 |
| March, - - - - | 4,260 | 1,934 | 10,796 | 2,907 | 6,096 |
| April, - - - - | 4,156 | 5,540 | 4,900 | 3,332 | 12,303 |
| May, - - - - | 4,295 | 3,602 | 5,909 | 4,152 | 3,183 |
| June, - - - - | 4,656 | 2,147 | 7,996 | 1,162 | 6,372 |
| July, - - - - | 4,155 | 5,463 | 3,878 | 4,844 | 3,739 |
| August, - - - | 4,567 | 6,653 | 2,494 | 7,060 | 10,256 |
| September, - - | 0,830 | 2,216 | 2,037 | 4,026 | 2,136 |
| October, - - - | 1,651 | 4,711 | 3,820 | 5,849 | 1,667 |
| November, - - | 3,602 | 2,272 | 2,905 | 3,046 | 4,960 |
| December, - - | 2,217 | 5,567 | 4,605 | 7,620 | 1,523 |
| Total, - - - | 43,441 | 47,332 | 67,664 | 63,240 | 60,276 |

| | 1836 | 1837 | 1838 | 1839 |
|---|---|---|---|---|
| January, - - - - - - | 4,848 | 1,523 | 5,226 | 2,631 |
| February, - - - - - | 3,048 | 4,107 | 2,869 | 2,076 |
| March, - - - - - - | 5,817 | 5,316 | 3,185 | 4,001 |
| April, - - - - - - - | 5,162 | 3,323 | 2,769 | 3,739 |
| May, - - - - - - - | 6,532 | 2,492 | 4,017 | 1,937 |
| June, - - - - - - - | 3,605 | 7,029 | 6,085 | 5,541 |
| July, - - - - - - - | 8,403 | 1,662 | 3,948 | 2,636 |
| August, - - - - - - | 6,129 | 5,546 | 0,692 | 1,800 |
| September, - - - - - | 1,246 | 4,015 | 3,190 | 2,221 |
| October, - - - - - - | 2,115 | 5,230 | 1,800 | 0,000 |
| November, - - - - - | 1,384 | 3,052 | 9,124 | 0,227 |
| December, - - - - - | 6,364 | 3,879 | 5,123 | 2,215 |
| Total, - - - - - - | 54,754 | 47,084 | 48,328 | 29,074 |

The average fall of rain for each of the last nine years has been fifty-one inches and one hundred and thirty-two thousands, (51,132.) It is interesting to compare this with the average of eleven years observations made at thirty-four different stations in the state of New York. As published in the American Almanac it is 35,150. The difference of latitude being 8 deg. and 15 sec. will give 1,938 as the increase in the fall of rain for every 69½ miles due south.

*Southern Advocate.*

